# Advances in Diagnostic Techniques for Influenza Virus Infection: A Comprehensive Review

**DOI:** 10.3390/tropicalmed10060152

**Published:** 2025-05-28

**Authors:** Qi Qian, Guohao Fan, Wei Yang, Chenguang Shen, Yang Yang, Yingxia Liu, Weiwei Xiao

**Affiliations:** 1 School of Public Health, Guangdong Medical University, Dongguan 523808, China; 18856226213@163.com; 2 Key Laboratory of Infectious Diseases Research in South China, Southern Medical University, Ministry of Education, Guangzhou 510515, China; a124965468@smu.edu.cn; 3 Shenzhen Key Laboratory of Pathogen and Immunity, Shenzhen Clinical Research Center for Infectious Disease, State Key Discipline of Infectious Disease, Shenzhen Third People’s Hospital, Second Hospital Affiliated to Southern University of Science and Technology, Shenzhen 518112, China; fgh15736988109@163.com (G.F.); yangwei19@mails.ucas.ac.cn (W.Y.); young@mail.sustech.edu.cn (Y.Y.)

**Keywords:** influenza virus, diagnostics, immunological tests, nucleic acid diagnosis, novel technologies

## Abstract

Influenza poses a significant global health burden due to its high transmissibility, antigenic variability, and substantial morbidity. The severe acute respiratory syndrome coronavirus 2 (SARS-CoV-2) pandemic has further complicated influenza dynamics, highlighting the need for rapid, accurate, and accessible diagnostics. This review comprehensively summarized the advancements in influenza virus (IFV) detection, from conventional methods like viral culture and serology to modern molecular techniques, including CRISPR-based systems, next-generation sequencing (NGS), and biosensors. We analyze the sensitivity, specificity, and applicability of these methods and emphasize their roles in clinical and public health settings. While traditional techniques remain valuable for strain characterization, novel technologies like CRISPR and portable biosensors offer rapid, low-resource solutions. This review provides a comprehensive insight into the development of integrated diagnostic strategies for seasonal IFV epidemics and future pandemics.

## 1. Introduction

Influenza is an acute respiratory infectious disease caused by the influenza virus (IFV). It is highly contagious, spreads rapidly, and is universally susceptible among the population [1]. IFV belongs to the family Orthomyxoviridae and is a single-stranded negative-sense RNA virus [2]. Currently, naturally occurring IFVs are classified into four types, namely Influenza A virus (IAV), Influenza B virus (IBV), Influenza C virus (ICV), and Influenza D virus (IDV) [1]. Among these, IAV and IBV are the main causes of human influenza epidemics, usually showing a seasonal peak in winter. Influenza can affect various organs in the human body. Since the virus is not confined to the respiratory system but can also trigger multisystem responses through systemic infection, the clinical manifestations of this disease are primarily characterized by acute fever, often accompanied by systemic and respiratory symptoms such as fever, chills, headache, cough, nasal congestion, rhinorrhea, sore throat, fatigue, and myalgia [3]. The occurrence of these symptoms is closely related to the virus’s invasion of multiple organs, particularly as the immune system releases inflammatory mediators to combat the virus, which can further exacerbate systemic discomfort. Relevant data show that influenza causes high morbidity and mortality globally. Approximately 10% of the global population is affected by influenza each year, with the number of deaths resulting from influenza infections ranging from 290,000 to 650,000 annually [4,5].

The emergence of the SARS-CoV-2 pandemic in 2019 resulted in altering the transmission dynamics of seasonal IFV [6]. Rapid, timely, and accurate diagnosis of IFV is of utmost importance for its control and treatment. Currently, the detection methods for IFV include virus isolation and culture, antigen detection, nucleic acid detection, and serological testing. In recent years, with the rapid development of biotechnology, such as gene sequencing, isothermal amplification techniques, and CRISPR technology, significant progress has been made in the IFV detection. However, existing detection methods still have some limitations and cannot fully meet the requirements of clinical practice and public health. Therefore, the development of more rapid, simple, sensitive, and specific detection methods for IFV remains an important research direction.

**Figure 1:**
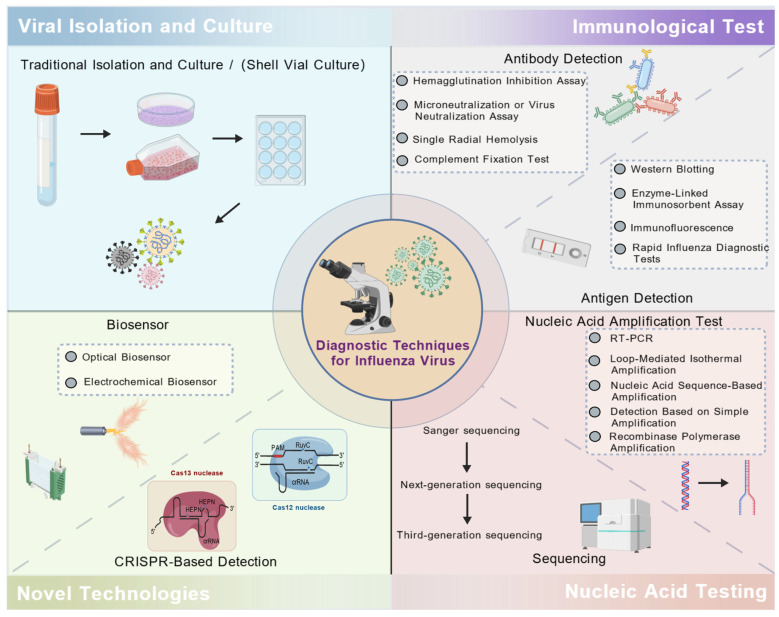
Laboratory and clinical tests for the detection of influenza virus.

This review aims to summarize the detection methods for IFV in terms of virus culture and isolation, immunological detection techniques, nucleic acid diagnostic techniques, and novel technologies (Figure 1), and discuss their respective advantages and disadvantages, as well as providing new perspectives for the development of such detection methods.

## 2. Viral Isolation and Culture

Viral isolation and culture were first established in the 1940s and have long been the fundamental methods for detecting IFV [7]. This method involves inoculating IFV from clinical samples into animal cells or embryonated eggs for propagation. Subsequently, the cytopathic effect (CPE) is observed, and methods such as hemagglutination assays, specific antibody staining, and immunofluorescence microscopy are used to confirm IFV infection [8,9]. The principle is to inoculate infectious samples into appropriate cell lines or embryonated eggs, followed by incubation for 7–10 days [9]. Viral culture is typically performed in established cell lines such as chicken embryo cells, Madin-Darby canine kidney (MDCK) cells, A549 cells, rhesus monkey kidney (LLC MK2) cells, buffalo green monkey kidney (BGMK) cells, rhesus monkey kidney (RhMK) cells, or African green monkey kidney (AGMK) cells. The CPE observed after inoculation varies depending on the cell type [9,10]. Although time-consuming, this approach is regarded as the gold standard for detecting IFV [10]. Relevant studies have shown that the specificity of this method reaches 100%, while the sensitivity varies depending on the cell line used, with the sensitivity of MDCK cells being 75.45% [11].

In consideration of the rapid mutation and high infectivity of IFV, the shell vial culture (SVC) method was introduced in the 1990s to improve the efficiency and convenience of IFV detection [12,13]. The SVC method involves inoculating test samples into cell cultures in small bottles or shell vials, followed by an incubation of 1–4 days. Confirmation of infection is then undertaken using immunofluorescence staining [14]. The SVC is usually performed in various cell cultures, including those of RhMK, MDCK, buffalo green monkey (BGM), human epidermoid carcinoma (HEp-2) cells, and fetal lung fibroblast (MRC-5) cells [13]. The SVC method has been shown to be faster and more sensitive than traditional culture methods, with virus detection possible within 1–2 days [15]. Subsequently, an improved SVC method using R-mix cells (a combination of mink lung cells and human adenocarcinoma cells) was established based on the SVC method [16]. This method combines two cell lines in a single shell vial, reducing the number of shell vials required. Most studies have found that the sensitivity of R-mix cells for detecting IAV and IBV are 89–98% and 94.7–100%, respectively [15,17]. Moreover, combining SVC with specific monoclonal antibodies can enhance the sensitivity and specificity of the assay while reducing turnaround time [18].

Viral isolation and culture offer crucial data for IFV strain genetic evolution and drug resistance analysis, yet they are limited by long cell separation cycles and high operational demands, making them unsuitable for rapid clinical diagnosis [19]. However, cell culture plays an important role in subsequent drug and vaccine research, evaluation, and study of the mechanism of viral infection, and is an indispensable method for IFV detection.

## 3. Immunological Test

Immunological test methods are more cost-effective and convenient when compared to virus culture methods and can be used for retrospective diagnosis as well as assessment of immune status. However, they generally have lower sensitivity and specificity compared to the culture methods [20]. These methods mainly include hemagglutination inhibition assay (HAI), microneutralization/virus neutralization assay (VN), single radial hemolysis (SRH), complement fixation test (CFT), enzyme-linked immunosorbent assay (ELISA), Western blotting (WB), Immunofluorescence (IF), and Rapid Influenza Diagnostic Tests (RIDTs) Method, and some of the characteristics of these immunological assays are summarized and compared (Table S1). Since routine serological testing for IFV requires paired samples collected at two different stages of infection (the acute phase and the convalescent phase), it often requires sequential sampling and is less suitable for immediate clinical decision-making [21].

### 3.1. Hemagglutination Inhibition Assay (HAI)

The HAI assay for IFV was first reported in the early 1940s as a method for quantifying IFV antibodies [22]. HAI assay is frequently utilized to detect IFV antibodies that inhibit the interaction between IFV hemagglutinin (HA) and receptors on red blood cells or cultured cells [23]. Additionally, it can be used to characterize antigenic differences among IFVs [24]. The principle of this method is that HA-specific antibodies can prevent influenza virus (approximately four hemagglutination units, primarily involving inactivated viruses) from binding to red blood cells derived from chickens, turkeys, humans, horses, or guinea pigs. A fourfold or greater increase in specific antibody titre is considered a positive indicator. The change in serum sample titres during the acute and convalescent phases is measured by hemagglutination inhibition [23]. The study by Sawant et al. demonstrated that the HAI is highly precise, specific, and sensitive for H3N2 influenza virus and can effectively detect novel IFV strains [7]. The HAI is relatively simple to perform, requiring no complex equipment or conditions, making it suitable for large-scale sample testing. However, cross-reactions may occur in the assay [25].

### 3.2. Microneutralization or Virus Neutralization Assay (VN)

The VN assay is a technique used to measure virus-specific antibody induction after natural infection or vaccination and is often the primary method for determining antibody titres against seasonal or avian IFV strains [9]. This method involves neutralizing the virus with virus-specific antibodies to prevent cellular infection. The virus neutralization titre is defined as the reciprocal of the highest serum dilution at which viral infection is completely inhibited. The VN is applicable to all IFV subtypes and has higher detection sensitivity than the HAI. For example, studies by Krivitskaya V.Z. have shown that compared to HAI, VN is more sensitive in detecting antibodies against H1N1, H1N1pdm09, and H3N2 viruses in human sera [26]. However, since this method requires the use of infectious live viruses, enhanced safety measures are necessary during the testing process, making it unsuitable for routine diagnostics [23,27].

### 3.3. Single Radial Hemolysis (SRH)

The SRH assay is a method for measuring serum antibody levels based on the diffusion of antibodies in agar gel [28]. This method quantitatively determines antibodies by measuring the hemolytic zones induced by antibody-antigen complexes. The size of the hemolytic zone is proportional to the antibody concentration, and it does not require serum processing [29]. This method is commonly used for antibody detection after natural infections and vaccinations. Studies have shown that SRH assays can detect subtle differences in antibody levels, distinguish between closely related IFV strains, and are more sensitive than HAI for detecting IBV [30]. The advantages of SRH assays include the requirement for only small amounts of virus and serum, the ability to analyze a large number of serum samples simultaneously without preprocessing (except for complement inactivation), and the generation of unbiased results after overnight incubation [30].

### 3.4. Complement Fixation Test (CFT)

The CFT is an immunological assay used to determine the antibody titre against IFV and the response of antibodies against the internal nucleoprotein (NP) and Matrix proteins of IFV after vaccination or infection [31]. The CFT utilizes the activation and binding properties of the complement system to detect specific antigen-antibody complexes. The complement system, a vital component of the human immune system, plays a critical role in immune defense and inflammatory responses [32]. The test consists of two stages. First, heat-treated antiserum (to inactivate complement) is mixed with antigen and complement to initiate antigen-antibody reactions. Subsequently, red blood cells sensitized with anti-red blood cell antibodies are added. If sufficient antigen-antibody reactions occur in the first stage, thereby consuming the complement, red blood cell hemolysis will not occur in the second stage. Conversely, if the complement remains unconsumed, hemolysis will take place. The degree of hemolysis can be used to infer the binding affinity of antigen and antibody [33]. Relevant studies have shown that the CFT can effectively detect IFVs and distinguish between influenza caused by IAV and IBV [34]. However, due to its complex procedures, strict requirements, low sensitivity, and susceptibility to false results, this test is now rarely used and has largely been replaced by simpler methods [35,36]. Nevertheless, it still retains value in specific situations.

### 3.5. Enzyme-Linked Immunosorbent Assay (ELISA)

ELISA was first applied in the 1990s and is mainly used for disease diagnosis, which is characterized by its high specificity and sensitivity. The principle of this method involves adding specific antibodies to wells of an ELISA plate coated with IFV antigens. These antibodies recognize and bind to the viral antigens. After removing unbound antibodies, enzyme-labeled secondary antibodies that bind to the previously added specific antibodies are added. Subsequently, the enzyme substrate is added, and the enzyme catalyzes the substrate to produce a detectable color change. The intensity of the color change, measured by a photometer, is proportional to the amount of IFV antigen present. Based on the signal strength, the presence and relative quantity of IFV in the sample can be determined [37,38]. Depending on the detection principle and procedural design, ELISA can be classified into direct, indirect, sandwich, and competitive methods. Among these, the sandwich method is the most commonly used detection approach, suitable for detecting large molecules with two or more antigenic determinants [39]. Relevant studies have shown that the sensitivity of ELISA for detecting IAV and IBV is 8 to 64 times higher than that of CFT and/or HAI [40]. Although the method has relatively high sensitivity, it also has some drawbacks, such as the complexity of antibody labeling, the need for multiple operational steps, and the possibility of false-negative results [41].

### 3.6. Western Blotting (WB)

WB, first introduced in 1979, is an experimental technique used to detect specific proteins [42]. When applied to IFV detection, WB can identify and quantify specific proteins of IFV. The underlying principle of this method involves the separation of proteins extracted from IFV-infected cells or tissues by gel electrophoresis, typically polyacrylamide gel electrophoresis (PAGE), according to their molecular weight [42]. WB is a highly sensitive and specific detection technique with a wide range of applications, including studying protein expression in the context of IFV, virus identification, vaccine development, and disease surveillance. Through this technique, researchers can gain a deeper understanding of the biological characteristics and infection mechanisms of IFV. WB can also be combined with ELISA (or VN) to enhance the sensitivity of detecting antibodies against IFV. For example, studies by Rowe et al. have demonstrated that combining WB with ELISA or VN can effectively improve the sensitivity of detecting antibodies against H5N1 virus. In particular, the combination of WB and ELISA can achieve 100% sensitivity and specificity in children under 15 years old [43]. However, WB involves complex procedures that require precise control of experimental conditions, such as incubation time and reagent concentrations. Additionally, it is relatively expensive due to the complexity of its procedures. Moreover, incomplete transfer of proteins during the blotting process may lead to erroneous results [44].

### 3.7. Immunofluorescence (IF)

IF assays, also known as immunofluorescence assays, are techniques that involve binding fluorochromes to antibodies to form fluorescent antibodies, which then react with antigens in the sample. Antigen detection is achieved using a fluorescence microscope [45]. This method relies on fluorescently labeled specific antibodies or antigens: fluorochromes, which do not affect the activity of labeled components, are conjugated to antibodies or antigens to create fluorescent probes. The fundamental principle of IF assays lies in the high specificity of antigen-antibody reactions: when a fluorescently labeled antibody (or antigen) binds specifically to its target antigen (or antibody) in the sample, the complex emits a detectable fluorescent signal under a fluorescence microscope. This allows for the visualization and quantification of specific antigens or antibodies [46,47].

IF assays are mainly classified into two types: direct fluorescent antibody (DFA) and indirect fluorescent antibody (IFA) assays. DFA is time-saving and cost-effective. When used to detect IFV, DFA typically involves precipitating respiratory epithelial cells onto a slide and staining them with influenza-specific antibodies conjugated to fluorescent dyes. This test can be completed within 1–4 h [48]. However, DFA is a manual assay; interpreting its results requires a high level of expertise and judgment. It also cannot be used to subtype IAV [49]. As a result, the sensitivity and specificity of DFA can vary significantly between laboratories [50]. IFA, on the other hand, uses an unlabeled primary antibody to bind to the antigen, followed by a fluorescently labeled secondary antibody that binds to the primary antibody to achieve virus detection [45]. Studies by Noyola D E et al. have shown that IFA can achieve a specificity of up to 97% when detecting IFV, effectively distinguishing between IAV and IBV [51]. IF assays are characterized by high specificity (up to 97%) and the ability to distinguish between IAV and IBV, with results typically available within 2–4 h [51]. They are suitable for rapid clinical diagnosis, but their sensitivity is relatively low.

### 3.8. Rapid Influenza Diagnostic Tests (RIDTs) Method

RIDTs are antigen-based assays designed for point-of-care testing (POCT), enabling swift diagnosis of IFV infections [52]. Monoclonal antibodies targeting the NP of the IFV are commonly used, and these antibodies are labeled with colloidal gold or colored latex particles. The assays are based on enzyme-linked immunosorbent analysis or immunochromatography. The products are usually available in the form of test strips, cassettes, or cards, and the detection can be completed within 30 min. Results can be intuitively observed based on color changes or other optical signals [52]. The tests are easy to perform, and their sensitivity and specificity have increased with continuous improvements, both reaching above 85% [53]. Currently, most RIDT products approved by relevant authorities can detect and differentiate between IAV and IBV [54]. During the peak of influenza activity, the positive predictive value of RIDTs is very high, while during non-influenza seasons, their negative predictive value is relatively high. The sensitivity of RIDTs is limited by viral antigenic variation, sample quality, and the inherent limitations of the method [53]. Although RIDTs are not highly sensitive for detecting influenza and often underperform when it comes to identifying novel and mutated strains of IAV, they remain a commonly used method in most clinical virology laboratories due to their fast result turnaround, simple testing procedures, and relatively low cost. The sensitivity of rapid influenza diagnostic tests for detecting seasonal IFV varies depending on the commercial kit [55].

## 4. Nucleic Acid Testing (NAT)

NAT primarily relies on PCR technology and virus-specific genetic material (DNA or RNA). NAT has higher sensitivity and specificity than serological testing, does not involve viral antigens or antibodies, and can serve as an early-stage clinical diagnostic tool for diseases [9]. Common NAT methods for human IFV mainly include Nucleic Acid Sequence-Based Amplification (NASBA), Real-Time Reverse Transcription Polymerase Chain Reaction (RT-PCR), Loop-Mediated Isothermal Amplification (LAMP), Recombinase Polymerase Amplification (RPA), Transcription-Mediated Amplification (TMA), and Sequencing. Here, we will summarize and compare some of the features of these NAT methods (Table S1).

### 4.1. Real-Time Reverse Transcription Polymerase Chain Reaction (RT-PCR)

RT-PCR, developed in the 1980s, represents the highest level of sensitivity and specificity among traditional detection methods, making it the preferred method for detecting IFV [56]. The procedure mainly consists of three steps: viral RNA extraction, reverse transcription, and amplification detection using fluorescence [57]. Intercalating dyes are relatively inexpensive and emit fluorescence when bound to double-stranded DNA amplified in the reaction. Commonly used dyes include SYBR green and methylene blue. However, they lack specificity and may lead to false-positive results [58]. Fluorescent probes, which are more costly, primarily bind to specific DNA sequences. Hydrolysis probes and molecular beacons (MBs) are the two most commonly used probes in influenza detection [59]. Hydrolysis probes offer high specificity, low background fluorescence, and the ability to perform multiplex detection with multiple fluorophores simultaneously [60]. MBs not only share the advantages of hydrolysis probes but also have the ability to distinguish alleles. However, due to the stringent physical requirements of MBs, their correct design and implementation can be challenging. Unlike the separation through degradation in hydrolysis probes, the fluorescent and quencher groups in MBs are separated through displacement during amplification [61]. Compared to cell culture and ELISA, RT-PCR is nearly 100 times more sensitive. However, it has some drawbacks, such as a long reaction time of approximately 1–8 h and relatively high costs [8]. Multiplex detection can reduce costs by identifying multiple viruses or viral subtypes in a single sample [62]. Additionally, the design of micro volume PCR relatively reduces the time and cost of traditional RT-PCR detection, mainly achieved by reducing the reagent volume from 20 µL to 5 µL [63].

### 4.2. Loop-Mediated Isothermal Amplification (LAMP)

LAMP is a nucleic acid amplification-based detection technology that enables the rapid and efficient amplification of target nucleic acid sequences under isothermal conditions (typically at 60–65 °C) [64]. Unlike traditional PCR, LAMP requires both RNA reverse transcriptase (for RNA viruses) and/or DNA polymerase, along with at least two sets of primers (outer and inner primers) to recognize six distinct regions in the viral cDNA [65]. Optional loop primers can further accelerate amplification by targeting additional regions, but the core design relies on outer and inner primer pairs for sequence-specific hybridization and exponential amplification. For RNA virus detection, the reaction mixture explicitly includes both reverse transcriptase (to convert RNA to cDNA) and DNA polymerase [66]. The DNA concentration is quantitatively analyzed using an intercalating dye. This method exhibits a higher sensitivity comparable to RT-PCR [67]. LAMP can also be used for multiplex detection, with solid-phase reagents facilitating IFV subtype detection in a reaction volume of just 1 µL [68]. The combination of microfluidic devices with LAMP allows for the detection of H1N1 within 30 min, achieving a sensitivity of 90.90% [69]. Under isothermal conditions, LAMP requires only a heating block for amplification, making it cost-effective. However, the design of primers remains a complex and time-consuming task that demands a substantial amount of relevant knowledge.

### 4.3. Nucleic Acid Sequence-Based Amplification (NASBA)

NASBA utilizes three enzymes, T7 RNA polymerase, avian myeloblastosis virus reverse transcriptase (AMV-RT), and RNase H, for real-time amplification of target RNA [70]. The process is initiated with a forward primer that targets the T7 promoter region, binding to the target RNA and allowing reverse transcriptase, which extends the target sequence. Subsequently, RNase H degrades the original RNA. A second primer then binds to the newly amplified product, and the reverse transcriptase extends this sequence. T7 RNA polymerase binds to the extended product and synthesizes new RNA. This amplification cycle is repeated until detectable RNA is produced [71]. NASBA is suitable for seasonal influenza and exhibits high sensitivity [72]. For instance, in studies targeting the novel swine-origin influenza A (H1N1) virus, NASBA has demonstrated a sensitivity, specificity, positive predictive value, and negative predictive value of 100% [73]. Additionally, NASBA presents several advantages, including low cost, simple operation, and no requirement for complex thermal cycling equipment, making it suitable for rapid on-site detection and capable of delivering results quickly [74]. However, the method also has limitations, including complex primer design and a higher rate of false positives [74].

Additionally, Transcription-Mediated Amplification (TMA), an isothermal RNA amplification method highly similar in principle to NASBA, can also be used for influenza virus detection [75]. This technique employs RNA polymerase and reverse transcriptase in coordination under isothermal conditions to achieve specific amplification of RNA targets. Similar to NASBA, the process begins with reverse transcriptase converting viral RNA into cDNA, followed by RNA polymerase transcribing the cDNA template to generate numerous RNA copies. Detection of these amplified products enables efficient identification of influenza viruses [76].

### 4.4. Detection Based on Simple Amplification (SAMBA)

SAMBA is based on NASBA. After the completion of amplification via NASBA, the results are visualized using nitrocellulose paper strips. The detection process consists of three steps, viral RNA extraction, DNA amplification, and detection utilizing a paper-based system [8]. This method exhibits high sensitivity and specificity, making it suitable for the detection of avian influenza and human seasonal influenza [77]. For instance, a study by Wu L T indicated that the sensitivity of SAMBA for detecting the pandemic H1N1 virus reached 95.3%, while its specificity was 99.4% [77]. Additionally, another study demonstrated that SAMBA achieved clinical sensitivity and specificity of 100% and 97.9%, respectively, for IAV, along with both clinical sensitivity and specificity of 100% for IBV [78]. Compared to traditional detection methods, SAMBA is faster and has an easier operation, making it particularly suitable for POCT [79].

### 4.5. Recombinase Polymerase Amplification (RPA)

RPA, a novel isothermal nucleic acid amplification technology, was first reported by Piepenburg et al. in 2006 [80]. The core components of this technology include the recombinase T4 UvsX and Bacillus subtilis DNA polymerase I (Pol I), while the reaction system also requires amplification templates, primers, and various raw materials [81]. The basic reaction process is as follows: In the presence of ATP and polyethylene glycol, the recombinase UvsX binds to RPA primers to form a recombinase-primer complex. This complex identifies and inserts into the homologous sequence of double-stranded DNA templates, forming a D-loop structure and initiating strand displacement reactions. Single-stranded binding proteins stabilize the displaced template strand to prevent primer expulsion. After the recombinase dissociates from the complex, DNA polymerase binds to the 3′-OH end of the primer in the presence of dNTPs for strand elongation, generating new complementary strands. Repeating these steps achieves exponential amplification of the target region on the template [81,82,83].

RPA is extremely rapid, typically yielding detectable amplification products within approximately 20 min. These products can be visualized via conventional agarose gel electrophoresis. The technique exhibits a strong anti-interference capability, with detection results highly consistent with those of PCR [83]. Studies have shown that RPA demonstrates specificities of 100%, 100%, and 90% for detecting H1N1, H3N2, and IBV, respectively, with 100% sensitivity across all targets [84]. Thanks to its high sensitivity and specificity, short detection time, and no need for thermal cycling, this method is well-suited for POCT and resource-limited settings. However, it has drawbacks such as relatively high kit costs and the absence of specialized software for designing RPA-specific primers [82].

### 4.6. Sequencing

Sequencing technologies encompass Sanger sequencing, next-generation sequencing (NGS), and third-generation sequencing (TGS). Sanger sequencing, as a first-generation sequencing technology, is highly accurate and inexpensive. Sanger sequencing successfully completed the Human Genome Project [85]. However, it requires prior knowledge of the nucleic acid sequence and has low throughput, making it inadequate for detecting new and unknown pathogens [85].

NGS, also known as massively parallel sequencing, deep sequencing, or high-throughput DNA sequencing, is a powerful tool capable of sequencing multiple samples simultaneously. It can detect and identify various pathogens within a single sample and has demonstrated significant advantages in the detection of novel pathogens, viral typing, mutation identification, and assessment of infection clusters [86,87]. The two primary approaches for pathogen detection using NGS are whole genome sequencing (WGS) and metagenomic sequencing. WGS enhances identification capabilities by comprehensively analyzing the genomes of cultured pathogens, while metagenomic sequencing can directly identify potential pathogens from samples without the need for culturing [88]. Currently, three main NGS-based methods are used for sequencing the IFV genome: PCR amplicon sequencing, target enrichment sequencing, and metagenomic sequencing [89,90,91]. Relevant studies have shown that NGS can both identify IFV and detect mutations through WGS [92]. Additionally, research by Lee D H et al. has demonstrated that for IAV, NGS can perform deep sequencing to characterize viral populations, achieve efficient WGS, and identify viral variants using non-sequence-dependent methods [93]. This method can rapidly and reliably identify pathogens in samples without prior knowledge of the viral genome sequence or the need for specific primers. However, NGS also has its drawbacks, including short read lengths, expensive detection instruments, longer processing times, and the requirement for extensive bioinformatics analysis [94].

TGS is a novel sequencing technology, following Sanger sequencing and NGS. TGS, also known as long-read sequencing, has the significant advantage of generating long and ultra-long reads, with single reads reaching hundreds of thousands of bases, potentially close to one million base pairs [95]. Moreover, TGS eliminates the need for PCR amplification, thus addressing the requirement for viral amplification in traditional sequencing technologies [94].

## 5. Novel Technologies

### 5.1. CRISPR-Based Detection

CRISPR is a gene-editing technology that evolved as an immune mechanism in bacteria and archaea to defend against the invasion of viruses and other foreign genetic materials [96]. When viruses integrate their genes into the bacterial genome, the CRISPR-Cas system identifies and cleaves this foreign DNA [96]. Studies have shown that the CRISPR-Cas system can be combined with RPA and LAMP to amplify and detect specific DNA. Cas proteins cleave specific targets by recognizing sequences that are complementary to the CRISPR RNA (crRNA). DETECTR—DNA endonuclease-targeted CRISPR trans-reporter, a method combining Cas12a single-stranded DNase (ssDNase) activation with isothermal amplification, enables specific detection of RNA viruses [97,98]. During the detection of IFV, samples containing unextracted IAV and IBV are lysed by heating to inactivate nucleases. Reverse transcription and amplification are then performed using RT-RPA or RT-LAMP, with specific primer pairs targeting the matrix (M) and HA genes. The amplified products are subsequently detected using DETECTR, followed by analysis via fluorescence or lateral flow assays [99]. This method exhibits high specificity, achieving approximately 96% specificity for detecting IAV and IBV, with sensitivities of 85.07% and 94.87%, respectively [100]. Moreover, it does not require specialized equipment and can be used in POCT settings such as airports, schools, and local community hospitals [101].

### 5.2. Biosensor

A biosensor is an instrument designed to detect biological substances by converts their concentration into electrical signals. It typically comprises four key components: biorecognition element (biological analyte), sensor, amplifier, and processor. When biological components bind to receptors, such as enzyme-ligand, antigen-antibody, DNA, or RNA on the electrode surface, signals are generated in the form of electric current, heat, or gas. These signals are subsequently transformed into a readable format by a transducer [102,103]. Biosensors are characterized by their portability, rapidity, miniaturization, cost-effectiveness, and suitability for on-site detection.

#### 5.2.1. Optical Biosensor

Optical biosensors represent a class of analytical devices combining biorecognition elements with optical transduction systems, operating through quantifiable signal generation correlated with analyte concentration. These systems are widely regarded as the most fundamental biosensor configuration [104]. Current optical biosensing can be categorized into label-free and labeled modes. The label-free mode directly generates measurable signals by the interaction between the analyte and the sensor. In contrast, labeled sensing generates optical signals through colorimetric, fluorescent, or luminescent methods [105]. Common signals used in optical sensors include absorbance or reflection, fluorescence, scattering, and interference. Representative implementations encompass fluorescence-based systems, surface-enhanced Raman spectroscopy (SERS) platforms, and plasmonic resonance devices [106]. Fluorescence biosensors constitute the predominant implementation, exploiting characteristic spectral shifts in fluorophore emission profiles resulting from specific biomolecular interactions to achieve target quantification. Among various optical biosensing modalities, Surface Plasmon Resonance (SPR) has emerged as a prominent approach due to its exceptional sensitivity to nanoscale refractive index variations. This technology provides a label-free, highly sensitive platform for real-time analysis of biomarker detection by monitoring the refractive index changes caused by molecular binding [107]. Relevant studies have shown that SPR biosensors have significantly improved the detection sensitivity for H5N1 antibodies, achieving detection limit of 193.3 ng/mL [108]. Additionally, research by Zhang Y et al. introduced a colorimetric biosensor based on dual-functional gold nanoparticles (AuNPs) for rapid influenza virus detection, exhibiting 20 min assay completion times with 10 nM sensitivity thresholds [109].

Despite these technological advancements, practical implementation faces persistent challenges, including device stability optimization, cost-effective manufacturing processes, and the requirement for enhanced user interface design with improved mobile data integration capabilities [110].

#### 5.2.2. Electrochemical Biosensor

Electrochemical biosensors detect pathogens by converting the chemical energy of the target pathogens into electrical energy. These biosensors can be classified into diverse types according to their detection principles and application methods, encompassing voltammetry, potentiometry, conductometry, amperometry, impedance methods, polarography, and capacitive and piezoelectric methods [111]. Moreover, depending on the incorporated biological elements, different kinds of electrochemical biosensors, such as immunosensors, DNA sensors, or microbial sensors can be fabricated [111].

Typically, electrochemical biosensors rely on enzyme-catalyzed reactions between immobilized biomolecules and target analytes. These reactions generate electrons, thereby affecting the electrical properties of the solution [112]. The impedance method, which has emerged in recent years, is capable of detecting minute changes occurring at the solution-electrode interface without requiring enzyme labeling of the sample. Consequently, it has also found applications in the detection of IFV [111]. In recent years, research by Yan J et al. has demonstrated that electrochemical DNA biosensors modified with nanomaterials can achieve dual-probe specific recognition and signal amplification. This type of biosensor can quantitatively detect the cDNA of IAV, with a detection range from 10 fM to 1 × 10^3^ nM and a detection limit of 5.42 fM. It also exhibits high specificity and selectivity [113]. Despite advantages including rapid detection, portability, high specificity, and good stability, these devices face limitations in long-term sensor longevity and cost-effectiveness [114].

## 6. Discussion

This review summarizes a variety of techniques in the field of influenza virus detection, including traditional viral culture and serological methods, molecular biological detection technologies, and emerging detection tools. Each detection method has its own characteristics and plays a unique role in different application scenarios. Traditional viral culture and serological methods are irreplaceable in aspects such as strain identification, virus tracing, and immune status assessment. However, these methods generally have a long detection cycle, making it difficult to meet the urgent needs of rapid clinical diagnosis and posing certain limitations in responding to sudden outbreaks.

In sharp contrast, molecular biological detection technologies have made significant progress. Representative techniques such as real-time reverse transcription polymerase chain reaction (RT-PCR) and loop-mediated isothermal amplification (LAMP) have shown remarkable performance in terms of detection speed and sensitivity. RT-PCR can achieve highly sensitive detection of viral nucleic acids in a relatively short period of time, providing strong support for early diagnosis. LAMP, with its isothermal amplification feature, does not require complex temperature control equipment and can rapidly and efficiently complete detection in resource-limited settings, significantly expanding the scope of detection applications. Therefore, molecular biological detection technologies have gradually become important tools for clinical diagnosis and epidemic monitoring, playing a key role in influenza prevention and control.

In recent years, emerging tools such as CRISPR technology and biosensors have shown great potential in influenza virus detection. CRISPR technology, which combines high specificity and rapid detection capabilities, can effectively detect specific sequences of influenza viruses [100]. Biosensors, with their portability and rapid detection capabilities, are suitable for on-site rapid testing [103]. In addition, optical and electrochemical biosensors have significant advantages in terms of detection speed and sensitivity, but their long-term stability and cost control still need further optimization [109,113]. Tables S1 and S2 provide a detailed summary of the sensitivity, specificity, detection time, and advantages and disadvantages of various detection methods. In practical applications, the choice of an appropriate detection method needs to consider the purpose of the test, the type of sample, laboratory conditions, and time requirements.

## 7. Conclusions

The evolution of influenza diagnostics has shifted from labor-intensive methods to rapid, molecular-based platforms. Traditional techniques like viral culture and serology remain important for surveillance but lack speed and scalability. NAT, particularly RT-PCR, sets the standard for sensitivity, while innovations like LAMP and CRISPR offer promising alternatives for decentralized settings. However, challenges persist. The sensitivity of rapid antigen detection varies and new technologies such as biosensors need to be improved. High-throughput sequencing holds potential for pathogen discovery and resistance monitoring but faces computationmyeloblastosisal and infrastructural hurdles. Future efforts should focus on integrating molecular precision with POCT practicality, leveraging AI and microfluidics for real-time analysis. Global standardization and equitable access to advanced diagnostics are essential for effective influenza management. As antigenic drift and zoonotic spillovers continue, a proactive, technology-driven approach is critical to mitigating influenza’s global impact.

## Supplementary Materials

The following supporting information can be downloaded at: https://www.mdpi.com/article/10.3390/tropicalmed10060152/s1, Table S1: Comparison of various immunologic diagnostic methods; Table S2:Comparison of nucleic acid testing methods.
