# Supplementary material for: Advances in Diagnostic Techniques for Influenza Virus Infection: A Comprehensive Review
Source: Trop Med Infect Dis. 2025 May 28;10(6):152. doi: 10.3390/tropicalmed10060152 (PMC12197469; doi:10.3390/tropicalmed10060152)
Supplement: Supplementary file 1 [file tropicalmed-10-00152-s001.zip › tropicalmed-3574104-supplementary.pdf]

Table 1. Comparison of various immunologic diagnostic methods

Detection method	Sensitivity	Specificity	Detection time	Advantages	Disadvantages	References
Hemagglutination Inhibition Assay	Moderate	High	4-6 h	Simple operation and suitability for large-scale sample testing	May cause cross-reactions	(Li et al., 2017) (Sawant et al., 2023) (Kaufmann et al., 2017)
Microneutralization or Virus Neutralization Assay	High	Very High	3-5 days	High sensitivity and specificity	Unsuitable for routine diagnostics	(Li et al., 2017) (Stephenson et al., 2009)
Single radial hemolysion	Moderate-High	High		Requires only small amounts of virus and serum	Relatively low sensitivity	(Trombetta et al., 2018) (Wang et al., 2017)
Complement fixation test	Low	Moderate	1-2 days	Detects recent infections	Complex procedures and low sensitivity	(Ding et al., 2023) (Nandakumar et al., 2025)
Enzyme-Linked Immunosorbent Assay	High	Moderate-High	3-6 h	High sensitivity	Complex antibody labeling, multiple operational steps, and the possibility of false-negative results	(Alhajj et al., 2023) (Tabatabaei & Ahmed, 2022) (Steininger et al., 2002)
Western Blotting	High	High	1-2 days	High sensitivity and specificity	Relatively expensive and complex procedures	(Rowe et al., 1999) (Kurien & Scofield, 2006)
Immunofluorescence Assay	Moderate-High	High	2-4 h	High specificity and simple operation	Low sensitivity	(Sawant et al., 2023) (Noyola et al., 2000)
Rapid Influenza Diagnostic Tests	High	High	30 min	Fast result turnaround, simple testing procedures, and relatively low cost	Low sensitivity	(Morehouse et al., 2022) (Control & Prevention, 2009)

Table 2. Comparison of nucleic acid testing methods

Detection method	Sensitivity	Specificity	Detection time	Advantages	Disadvantages	References
Viral isolation and culture	75.45%	100%	7-10 days	Gold standard	Time-consuming	(Vemula et al., 2016) (Kim & Poudel, 2013)
RT-PCR	84.4%	100%	1-8 h	High sensitivity and specificity	High detection cost and time-consuming	(Vemula et al., 2016) (Dziąbowska et al., 2018)
Loop-Mediated Isothermal Amplification	93.8%	100%	1-1.5 h	High sensitivity and specificity, cost-effective	Complex primer design process	(Mahony et al., 2013) (Abe et al., 2011)
Nucleic Acid Sequence-Based Amplification	100%	100%	2 h	Cost-effective and simple operation	Complex primer design process and higher rate of false positives	(Ge et al., 2010) (Morabito et al., 2013)
Detection based on Simple Amplification	95.3%-100%	97.9-100%	85 min	Faster and easier operation	Complex primer design process	(Wu et al., 2013) (Wu LiangTa et al., 2010) (Boora et al., 2024)
Recombinase Polymerase Amplification	100%	90%-100%	20 min	High sensitivity and specificity, short detection time, no need for thermal cycling	Relatively high kit costs	(Tan et al., 2022) (Liang et al., 2023) (Lobato & O'Sullivan, 2018)
Sequencing	-	-	-	High sensitivity and specificity, fast result turnaround	Short read lengths, expensive detection instruments, and longer processing times	(Lee, 2020) (Van Poelvoorde et al., 2020)
CRISPR-Based Detection	85.07%(IAV) 94.87%(IBV)	96%	1-2 h	High sensitivity and specificity, quick detection	Limited detection range	(Mayuramart et al., 2021) (Li & Ren, 2020)
Biosensor	-	-	20 min	High sensitivity and specificity, portability	Long-term sensor longevity and low stability optimization	(Bhalla et al., 2016) (Naresh & Lee, 2021)
